# Inhibition of N‐myc expression sensitizes human neuroblastoma IMR‐32 cells expressing caspase‐8 to TRAIL

**DOI:** 10.1111/cpr.12577

**Published:** 2019-02-06

**Authors:** Myoung Woo Lee, Dae Seong Kim, Hye Ryung Kim, Hyun Jin Park, Ji Won Lee, Ki Woong Sung, Hong Hoe Koo, Keon Hee Yoo

**Affiliations:** ^1^ Department of Pediatrics Samsung Medical Center, Sungkyunkwan University School of Medicine Seoul Korea; ^2^ Department of Health Sciences and Technology SAIHST, Sungkyunkwan University Seoul Korea

**Keywords:** caspase‐8, cisplatin, neuroblastoma, N‐myc, TRAIL

## Abstract

**Objectives:**

This study aims to explore the roles of N‐myc and caspase‐8 in TRAIL‐resistant IMR‐32 cells which exhibit *MYCN* oncogene amplification and lack caspase‐8 expression.

**Materials and methods:**

We established N‐myc–downregulated IMR‐32 cells using shRNA lentiviral particles targeting *N‐myc* and examined the effect the N‐myc inhibition on TRAIL susceptibility in human neuroblastoma IMR‐32 cells expressing caspase‐8.

**Results:**

Cisplatin treatment in IMR‐32 cells increased the expression of death receptor 5 (DR5; TRAIL‐R2), but not other receptors, via downregulation of NF‐κB activity. However, the cisplatin‐mediated increase in DR5 failed to induce cell death following TRAIL treatment. Furthermore, interferon (IFN)‐γ pretreatment increased caspase‐8 expression in IMR‐32 cells, but cisplatin failed to trigger TRAIL cytotoxicity. We downregulated N‐myc expression in IMR‐32 cells using N‐myc–targeting shRNA. These cells showed decreased growth rate and Bcl‐2 expression accompanied by a mild collapse in the mitochondrial membrane potential as compared with those treated with scrambled shRNA. TRAIL treatment in N‐myc–negative cells expressing caspase‐8 following IFN‐γ treatment significantly triggered apoptotic cell death. Concurrent treatment with cisplatin enhanced TRAIL‐mediated cytotoxicity, which was abrogated by an additional pretreatment with DR5:Fc chimera protein.

**Conclusions:**

N‐myc and caspase‐8 expressions are involved in TRAIL susceptibility in IMR‐32 cells, and the combination of treatment with cisplatin and TRAIL may serve as a promising strategy for the development of therapeutics against neuroblastoma that is controlled by N‐myc and caspase‐8 expression.

## 1 INTRODUCTION

Neuroblastoma is the most common extracranial solid tumour of childhood and accounts for 15% of cancer‐related deaths.^1^, ^2^ Despite recent advances in cancer treatment, aggressive neuroblastomas remain refractory to current therapy; the overall 5‐year survival rate for patients with advanced‐stage neuroblastoma is 30%‐40%.^2^ Amplification of *MYCN* oncogene is observed in approximately 20% of neuroblastomas and 45% of high‐risk cases.^3^ *MYCN* amplification is strongly associated with poor outcome^2^, ^4^ and has been considered as the most important prognostic factor,^5^ which strongly correlated with advanced‐stage disease and treatment failure. The deregulation of *MYCN* oncogene that regulates the expression of genes involved in several processes, including cell cycle,^6^, ^7^ proliferation,^8^, ^9^ differentiation^10^, ^11^ and apoptosis,^6^, ^8^, ^10^ is sufficient to drive the transformation of neural crest progenitor cells into neuroblastoma.

Tumour necrosis factor (TNF)–related apoptosis‐inducing ligand (TRAIL), also known as the Apo‐2 ligand, is a member of TNF ligand superfamily that selectively induces apoptosis in a wide variety of transformed cell lines from diverse tissue types.^12^ TRAIL may induce apoptosis through its interaction with two of four membrane‐bound receptors, namely death receptor 4 (DR4; TRAIL‐R1) and DR5 (TRAIL‐R2). These receptors bear a protein‐protein interaction motif termed as the “death domain (DD)”.^13^, ^14^ The other two receptors, decoy receptor 1 (DcR1; TRAIL‐R3) and DcR2 (TRAIL‐R4), either lack the cytoplasmic or truncated DD. TRAIL induces receptor trimerization and conformational change in the intracellular DD, resulting in the recruitment of Fas‐associated DD.^15^ This signals death through the formation of a death‐inducing signal complex, which rapidly activates caspase‐8. Caspase‐8 mediates apoptosis either through the direct activation of the downstream effector caspases or by the cleavage of pro‐apoptotic molecules such as B‐cell lymphoma 2 (Bcl‐2) homolog, Bid.^16^, ^17^

Studies have shown that anti‐cancer drugs such as bortezomib,^18^, ^19^ etoposide^20^ and doxorubicin^21^ sensitized cancer cells to TRAIL‐mediated death through the upregulation of DR expression. In particular, the upregulation of DRs by cisplatin affected TRAIL‐induced apoptosis in many cancer types, such as squamous carcinoma,^22^ hepatocellular carcinoma^23^ and colon cancer.^24^ The mechanism underlying the upregulation of TRAIL receptors is variable. The activation or inhibition of nuclear factor kappa B (NF‐κB)^20^, ^25^ and/or extracellular signal–regulated kinase (ERK) 1/2^26^, ^27^ may upregulate both DR4 and DR5, while p53 may mediate the upregulation of DR5 at transcriptional levels.^28^ In addition, chemotherapeutic agents may mediate the changes in the rate of receptor turnover at cell surface.^29^, ^30^

In this study, we investigated whether cisplatin treatment triggers TRAIL‐mediated cytotoxicity in TRAIL‐resistant IMR‐32 neuroblastoma cells which exhibit amplification of *MYCN* oncogene and lack caspase‐8 expression. Our data, for the first time, show that TRAIL susceptibility correlated with the expression levels of N‐myc and caspase‐8 in human neuroblastoma IMR‐32 cells. The combination therapy of cisplatin and TRAIL is a promising strategy for treating neuroblastoma that is controlled by the expression of N‐myc and caspase‐8, and its use may provide important information for the development of additional potential therapeutic strategies to fight neuroblastoma.

## 2 MATERIALS AND METHODS

### 2.1 Reagents

Cisplatin was purchased from Dong‐A Pharm (Seoul, Korea) and NF‐κB activation inhibitor from Calbiochem (Darmstadt, Germany). Human recombinant TRAIL, Alamar Blue^®^ and trypan blue were purchased from Life Technologies (Rockville, MD); interferon (IFN)‐γ, human recombinant DR5/Fc chimera (DR5:Fc) protein and phycoerythrin (PE)‐conjugated antibodies for DR4, DR5, DcR1 and DcR2, from R&D Systems (Minneapolis, MN); antibodies for N‐myc, Bid, p27^Kip1^, p21^Cip1/Waf1^, caspase‐3 and caspase‐9, from Cell Signaling Technology (Danvers, MA); and antibodies for caspase‐8, Bcl‐2, Bax, poly(ADP‐ribose) polymerase (PARP) and β‐actin, scrambled shRNA (Cat. No: sc‐108080) as well as *N‐myc* shRNA (Cat. No: sc‐36003‐V) lentiviral particles, and polybrene, from Santa Cruz Biotechnology (Santa Cruz, CA). Hoechst 33258 dye and puromycin were purchased from Sigma‐Aldrich (St. Louis, MO), and tetramethylrhodamine ethyl ester perchlorate (TMRE) was purchased from Thermo Fisher Scientific (Waltham, MA).

### 2.2 Cell viability: Alamar Blue assay

Human malignant neuroblastoma cell lines IMR‐32 and SK‐N‐BE, and neuroepithelioma cell line SK‐N‐MC were purchased from the American Type Culture Collection (ATCC; Manassas, VA). Details of the cell culture are described in Supporting information Methods. Cells (2 × 10^4^ cells/well) were seeded in a 96‐well plate (Nalgene Nunc, Naperville, IL), in 100 μL of Dulbecco's modified Eagle's medium (DMEM; Biowest, Nuaillé, France) containing 1% heat‐inactivated foetal bovine serum (FBS; Biowest) in the absence of phenol red, and treated for 24‐96 hours with either IFN‐γ, DR5:Fc, NF‐κB activation inhibitor, cisplatin or TRAIL, separately and in different combinations. Then, 3 hours before the end of the incubation, cells were treated with 11 μL of 1× Alamar Blue®, and their absorbance was measured at 570 and 600 nm wavelength with an enzyme‐linked immunosorbent assay (ELISA) reader (Molecular Devices, Sunnyvale, CA).

### 2.3 Flow cytometry

For the measurement of TRAIL receptor expression, 10^6^ cells were suspended in 0.1 mL of phosphate‐buffered saline (PBS; Biowest) and incubated with PE‐conjugated antibodies for 45 minutes at 4°C. PE‐conjugated human IgG (R&D Systems) was used as isotype control at concentrations similar to that of the specific antibodies. The fluorescence intensity of the samples was evaluated with fluorescence‐activated cell sorting (FACSCalibur; BD Bioscience, San Jose, CA), and data were analysed using CELLQUEST software (BD Bioscience).

### 2.4 Measurement of human NF‐κB (p50/p65) activity

The DNA‐binding activity of NF‐κB in cells was quantified by ELISA using NF‐κB p50 and p65 transcription factor assay kit (Cayman, Ann Arbor, MI) according to the manufacturer's instructions. Details of the measurement of NF‐κB activity are described in supplementary methods.

### 2.5 Immunoblotting

Cells were washed with cold PBS and lysed in cold radioimmunoprecipitation assay (RIPA) lysis buffer (Thermo Fisher Scientific) with a protease inhibitor cocktail (Thermo Fisher Scientific). Cell lysates were centrifuged at 3000 *g* for 15 minutes at 4°C. The supernatant was harvested and the protein concentration analysed using the BCA protein assay kit. For electrophoresis, 50 μg of protein was dissolved in the reduced sample buffer (Thermo Fisher Scientific) and separated on a NuPAGE 4%‐12% Bis‐Tris gel (Novex, Carlsbad, CA). Separated proteins were transferred onto polyvinylidene difluoride membranes (GE Healthcare, Buckinghamshire, UK). Blots were blocked for 1 hours in Tris‐buffered saline (TBS; Biosesang, Korea) containing 2% bovine serum albumin (Life Technologies—Gibco), washed thrice with 0.3% TBST (TBS containing 0.3% Tween 20 [Sigma‐Aldrich]) and incubated at 4°C overnight with primary antibodies (all antibodies, 1:1000 dilution) in 0.1% TBST. Blots were then washed thrice with 0.3% TBST and incubated for 1 hours with horseradish peroxidase–conjugated secondary antibodies (1:3000 dilution, Cell Signaling Technology) in 0.3% TBST. After washing thrice with 0.3% TBST, protein bands were visualized with an enhanced chemiluminescence detection system (GE Healthcare).

### 2.6 Luminex assay

Caspase‐3 activation and PARP cleavage were assessed using 3‐Plex Apoptosis Signaling Kit (Merck Millipore, St. Charles, MO) according to the manufacturer's instructions. Details of the Luminex assay are described in Supplementary methods.

### 2.7 Nuclear staining and phase‐contrast microscopy

Cells were treated with IFN‐γ, cisplatin and TRAIL, either separately or in different combinations. After treatment, cells were incubated with Hoechst 33258 at a final concentration of 5 μg/mL in HEPES buffer (Life Technologies‐Gibco) for 20 minutes at 37°C in a 5% CO_2_ incubator. Cells were resuspended in PBS and were examined with a Nikon Eclipse TE2000‐U inverted fluorescence microscope equipped with a Nikon LH‐M100C‐1 camera (Nikon Corporation Instruments Company, Japan).

### 2.8 Establishment of N‐myc–downregulated IMR‐32 cells

For the transduction of IMR‐32 cells with N‐myc–downregulating lentiviral particles, cells were pretreated with 5 μg/mL polybrene, prepared in DMEM containing 10% FBS and incubated with the lentivirus at a multiplicity of infection (MOI) of 5. After incubating for 24 hours at 37°C with 5% CO_2_, cells were washed twice with PBS and fresh medium was added. Infected cells were selected after 10 days of incubation in the medium containing 5 μg/mL puromycin, after 2 weeks of transduction. The protein level of N‐myc was confirmed by Western blot analysis.

### 2.9 Phase‐contrast microscopy and cell counting

Viable cells were incubated at 37°C with 5% CO_2_, and the medium was changed every 3‐4 days. Cells were observed on days 3, 7 and 14, using an inverted microscope (Olympus CK40, Melville, NY). Collected cells were resuspended in PBS and stained with trypan blue. The total number of viable cells was determined using a haemocytometer (Marienfeld, Germany).

### 2.10 Measurement of mitochondrial membrane potential: TMRE staining

The mitochondrial membrane potential (MMP, Ψm) was measured with the fluorescent dye TMRE. Cells were incubated with TMRE at a final concentration of 50 nmol/L in HEPES buffer for 20 minutes at 37°C in a 5% CO_2 _incubator. Cells were then washed with PBS and examined using a Nikon Eclipse TE2000‐U inverted fluorescence microscope equipped with a Nikon LH‐M100C‐1 camera.

### 2.11 Statistical analysis

All data were expressed as mean ± standard deviation (SD). Student's *t* test was used for statistical comparison between groups. A value of *P* < 0.05 was considered statistically significant.

## 3 RESULTS

### 3.1 Cisplatin increased DR5 expression in IMR‐32 cells via downregulation of NF‐κB activity

**Figure 1:**
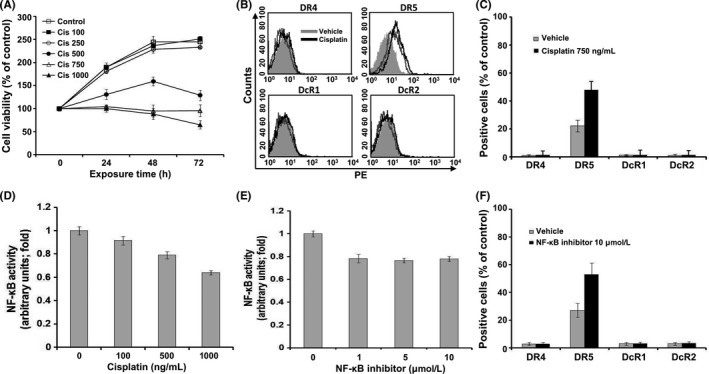
Cisplatin increased DR5 expression in human neuroblastoma IMR‐32 cells via downregulation of NF‐κB activity. A, Cell viability in response to treatment with 100, 250, 500, 750 and 1000 ng/mL cisplatin in human neuroblastoma IMR‐32 cells by Alamar Blue assay. Data are expressed as the percentage (mean ± SD) of vehicle‐treated control from three independent experiments. B, C, The expression of TRAIL receptors (DR4, DR5, DcR1 and DcR2) in IMR‐32 cells by flow cytometry using PE‐conjugated antibodies. Positive cells were plotted against the appropriate IgG isotype controls (shaded histogram). Data were obtained at 24 h after treatment with vehicle (unshaded grey line) or 750 ng/mL cisplatin (unshaded black line). Changes in the expression of TRAIL receptors in response to cisplatin are shown as (B) histogram and (C) bar graph. D, Cells treated with the indicated concentrations of cisplatin or E, NF‐κB inhibitor for 12 h. Nuclear extracts were prepared from these cells, and the DNA‐binding activity of NF‐κB was quantified by ELISA. Data are expressed as a fold change over vehicle‐treated control cells (mean ± SD) from two independent experiments. F, Changes in the expression of TRAIL receptors in response to treatment with NF‐κB inhibitor for 24 h. Data are expressed as a percentage of IgG isotype controls (mean ± SD) from three independent experiments. Cis, cisplatin

To examine whether cisplatin induces cell death in neuroblastoma IMR‐32 and SK‐N‐BE cell lines, cells were treated with 100‐1000 ng/mL cisplatin for the indicated time. Although low concentrations of cisplatin showed limited cytotoxic effect, a gradual increase in the death of neuroblastoma IMR‐32 (Figure 1A) and SK‐N‐BE cells (Figure S1A) was observed with an increase in cisplatin concentration. To elucidate the effect of cisplatin on the expression of TRAIL receptors, we examined the expression levels of TRAIL receptors in IMR‐32 and SK‐N‐BE cells after cisplatin treatment by flow cytometry. As shown in Figures 1B and C, only DR5 was expressed in IMR‐32 cells, and cisplatin induced the expression of DR5 in these cells. Unlike IMR‐32 cells, TRAIL receptors were detected at low levels in SK‐N‐BE cells, and cisplatin treatment increased the expression of all TRAIL receptors (Figures S1B and C). Moreover, cisplatin treatment significantly decreased NF‐κB activity in IMR‐32 cells in a dose‐dependent manner (Figure 1D). Consistent with these results, NF‐κB activation inhibitor decreased NF‐κB activities in IMR‐32 cells (Figure 1E), accompanied by an increase in DR5 expression (Figure 1F). We also established NF‐κB–downregulated IMR‐32 cells using shRNA‐targeting *NF‐κB p65* and observed that these cells showed increased expression of DR5, which was unaffected by cisplatin treatment (Figure S2). These results indicated that cisplatin treatment increased DR5 expression in IMR‐32 cells, via downregulation of NF‐κB activity.

### 3.2 Cisplatin failed to trigger TRAIL cytotoxicity in TRAIL‐resistant IMR‐32 cells

**Figure 2:**
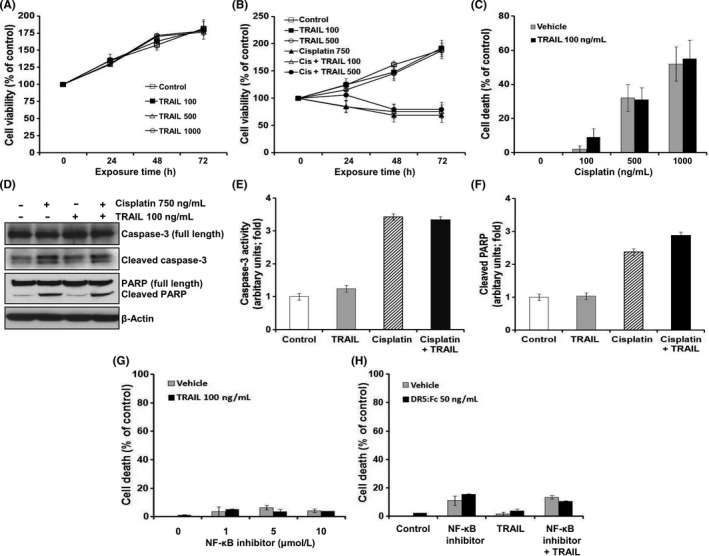
Cisplatin treatment failed to trigger TRAIL cytotoxicity in TRAIL‐resistant IMR‐32 cells. A, Cell viability in response to treatment with 100, 500 and 1000 ng/mL TRAIL with time. B, Cell viability in response to pretreatment with 750 ng/mL cisplatin for 12 h and consecutive treatment with 100 or 500 ng/mL TRAIL with time. C, Cell death in response to pretreatment with either 100, 500 or 1000 ng/mL cisplatin for 12 h and consecutive treatment with 100 ng/mL TRAIL for 36 h. D‐F, Cells pretreated with 750 ng/mL cisplatin for 12 h, followed by 100 ng/mL TRAIL treatment for 24 h. D, Cleavage of caspase‐3 and PARP in cell lysates by immunoblotting. β‐Actin was used as an internal control. Cell lysates were analysed for (E) activated caspase‐3 and (F) cleaved PARP using Luminex assay. G, Cell death in response to pretreatment with either 1, 5 or 10 μmol/L NF‐κB inhibitor for 12 h and consecutive treatment with 100 ng/mL TRAIL for 36 h. H, Cell death in response to treatment with 10 μmol/L NF‐κB inhibitor and/or 500 ng/mL TRAIL for 48 h in the absence or presence of 50 ng/mL DR5:Fc by Alamar Blue assay. Data are expressed as the percentage (mean ± SD) of vehicle‐treated control cells from three independent experiments. Cis, cisplatin

We measured the susceptibility of human neuroblastoma IMR‐32 and SK‐N‐BE cells to TRAIL with Alamar Blue assay. TRAIL treatment had no cytotoxic effect on both cells (Figures 2A and S3A). As described above, cisplatin treatment increased TRAIL receptors, particularly DR5, only in IMR‐32 cells. To elucidate the effect of increased TRAIL receptors on cytotoxicity, we analysed TRAIL cytotoxicity in IMR‐32 and SK‐N‐BE cells pretreated with cisplatin. As shown in Figures 2B and C, pretreatment with cisplatin failed to increase TRAIL cytotoxicity in IMR‐32 and SK‐N‐BE cells (Figure S3B). No difference in the viability was observed between cells pretreated with cisplatin for 12 hours before TRAIL treatment and those treated with cisplatin alone. Although cisplatin treatment induced caspase‐3 and PARP cleavage, an indication of apoptotic cell death, the combination of cisplatin and TRAIL failed to alter caspase‐3 and PARP cleavage in IMR‐32 cells (Figure 2D). Similar observations related to caspase‐3 and PARP cleavage were also recorded in the Luminex assay, indicating that cisplatin failed to induce TRAIL cytotoxicity in IMR‐32 cells (Figure 2E and F). Moreover, no difference was observed in the viability of IMR‐32 cells pretreated with NF‐κB activation inhibitor for 12 hours before TRAIL and those treated with NF‐κB activation inhibitor alone (Figure 2G and H). These results indicate that cisplatin treatment failed to trigger TRAIL cytotoxicity in IMR‐32 cells, although it increased DR5 expression in these cells via downregulation of NF‐κB activity.

### 3.3 Interferon‐γ restored caspase‐8 expression in IMR‐32 cells but failed to induce TRAIL cytotoxicity

**Figure 3:**
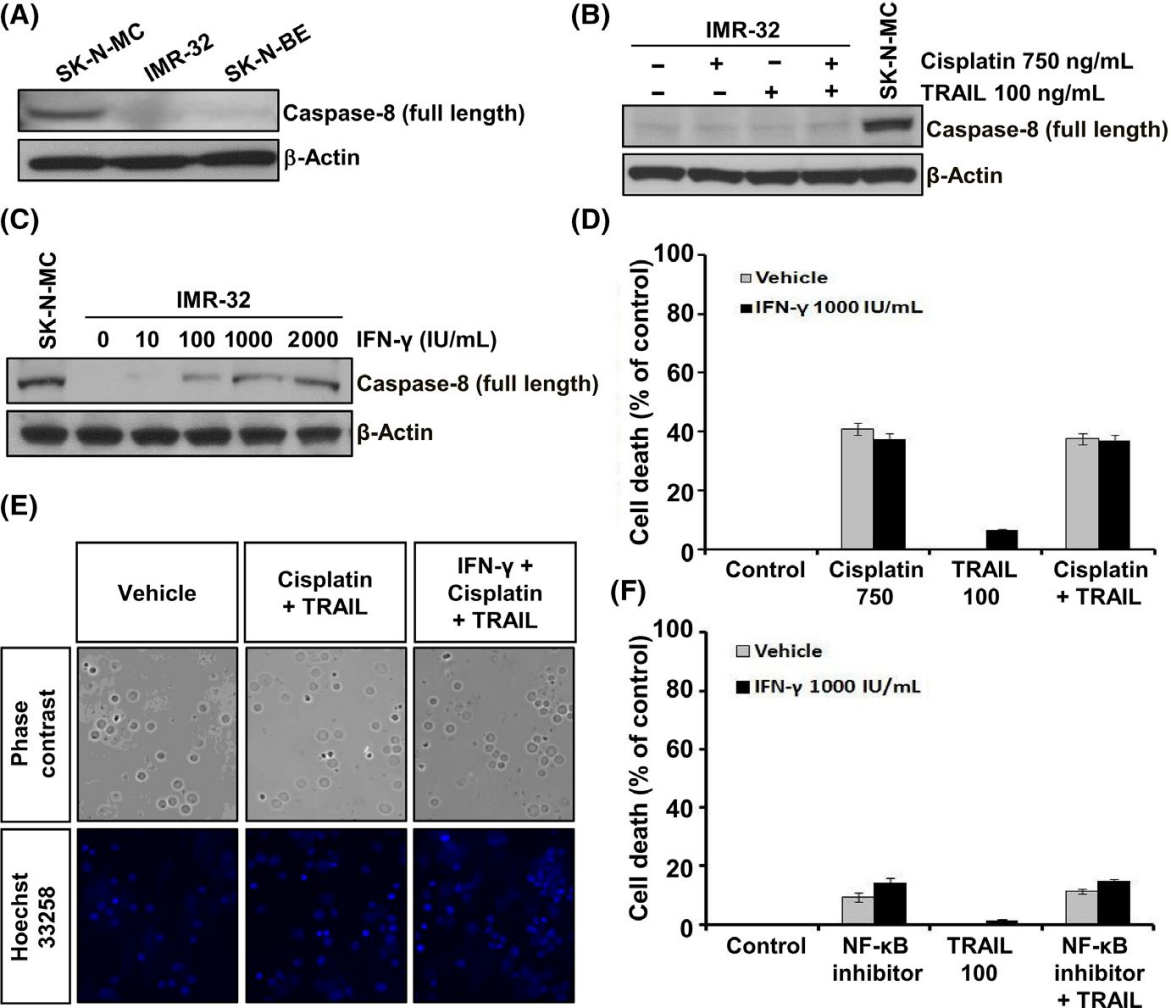
Interferon‐γ treatment restored caspase‐8 expression but failed to induce TRAIL cytotoxicity in IMR‐32 cells. A, Expression levels of caspase‐8 in SK‐N‐MC (positive control) and neuroblastoma IMR‐32 and SK‐N‐BE cells. B, Analysis of caspase‐8 expression in IMR‐32 cells treated with 750 ng/mL cisplatin and/or 100 ng/mL TRAIL for 24 h. C, Restoration of caspase‐8 expression in IMR‐32 cells, with SK‐N‐MC cells, was used as a positive control, following treatment with indicated concentrations of IFN‐γ for 24 h by immunoblotting. β‐Actin was used as an internal control. D, Assessment of IMR‐32 cells death when exposed to 1000 IU/mL IFN‐γ for 24 h, followed by treatment with 750 ng/mL cisplatin and/or 100 ng/mL TRAIL for 48 h, by Alamar Blue assay. E, Representative images of these cells stained with Hoechst 33258. F, IMR‐32 cells exposed to 1000 IU/mL IFN‐γ for 24 h, followed by treatment with 10 μM NF‐κB inhibitor and/or 100 ng/mL TRAIL for 48 h. Data are expressed as the percentage (mean ± SD) of vehicle‐treated control cells from three independent experiments. IFN‐γ, interferon‐γ

**Figure 4:**
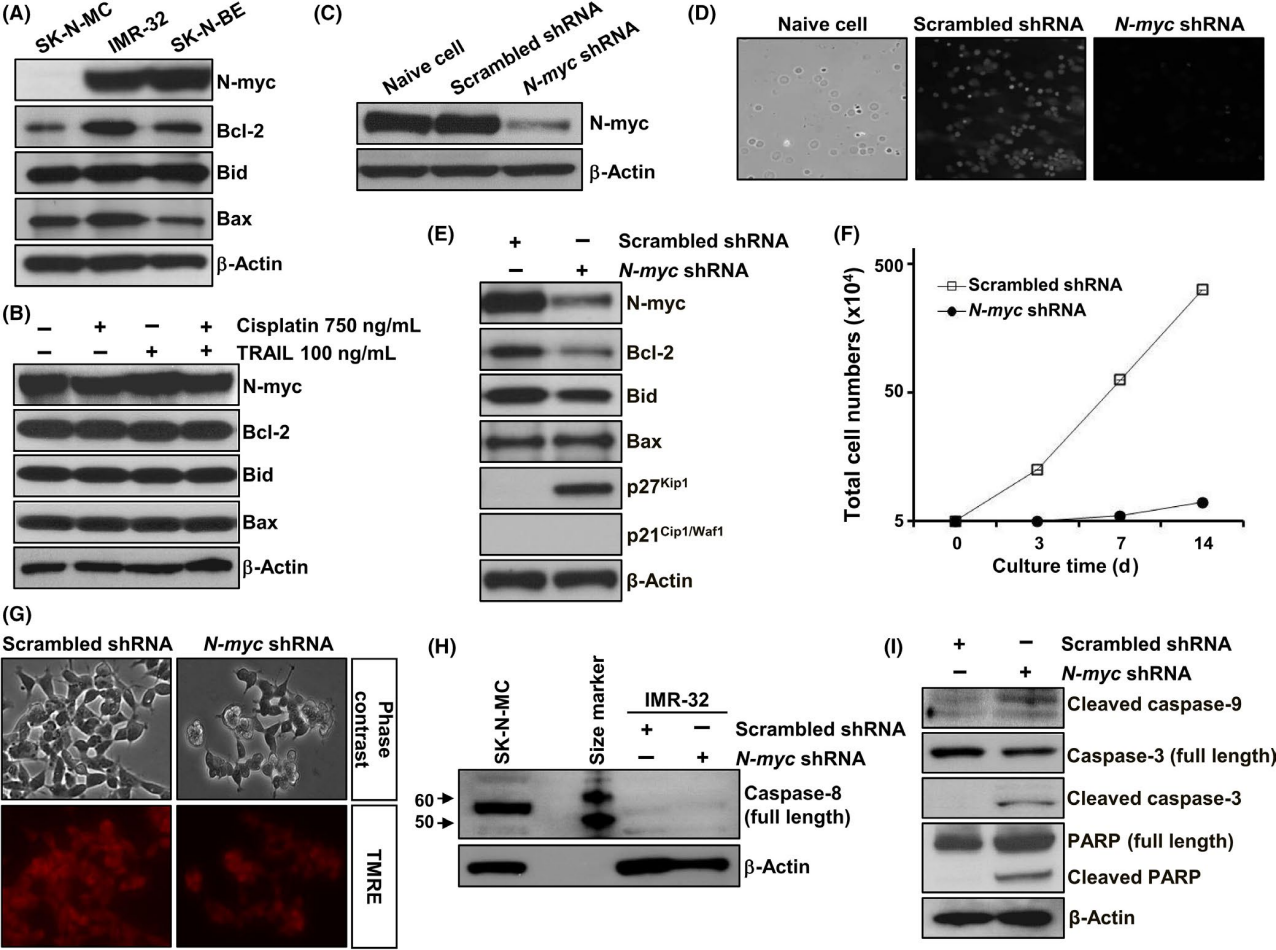
The change in the molecular and cellular characteristics of N‐myc–downregulated IMR‐32 cells. A, Expression level of N‐myc and Bcl‐2 family (Bcl‐2, Bid and Bax) in SK‐N‐MC, IMR‐32 and SK‐N‐BE cells determined by immunoblotting. B, Expression level of N‐myc, Bcl‐2, Bid and Bax in IMR‐32 cells after treatment with 750 ng/mL cisplatin and/or 100 ng/mL TRAIL for 24 h. C, Expression level of N‐myc in IMR‐32 cells infected with lentivirus carrying shRNA‐targeting N‐myc. D, Phase‐contrast images of these cells. E, Expression level of N‐myc, Bcl‐2, Bid, Bax, p27^Kip1^ and p21^Cip1/Waf1 ^in N‐myc–downregulated IMR‐32 cells determined by immunoblotting. F, Total number of viable cells determined by trypan blue exclusion method using a hemocytometer. G, Measurement of mitochondrial membrane potential (Ψm) in N‐myc–downregulated IMR‐32 cells by TMRE staining. H, I, Analysis of caspase‐8 expression and caspase‐9, caspase‐3 and PARP cleavage in N‐myc–downregulated IMR‐32 cells by immunoblotting. H, Caspase‐8 expression was undetected in N‐myc–downregulated IMR‐32 cells. SK‐N‐MC cells were used as a positive control. I, Caspase‐3, caspase‐9 and PARP were slightly cleaved in N‐myc–downregulated IMR‐32 cells. β‐Actin was used as an internal control. Representative immunoblots are shown

In contrast to SK‐N‐MC cells used as a positive control, IMR‐32 and SK‐N‐BE cells were negative for caspase‐8 expression (Figure 3A), which was unaltered by cisplatin and/or TRAIL treatment for 24 hours (Figure 3B). To restore the expression of caspase‐8 in IMR‐32 cells, we treated these cells with a demethylating agent, IFN‐γ or 5‐azacytidine (5‐AzaC), for 24 hours and found that the caspase‐8 expression was significantly restored (Figures 3C and 4A). 5‐AzaC treatment slightly induced cell death (Figure S4B), but pretreatment with 5‐AzaC and/or IFN‐γ failed to trigger TRAIL cytotoxicity in IMR‐32 cells (Figure S4B and C). Although caspase‐8 expression was restored in IMR‐32 cells exposed to IFN‐γ, treatment of these cells with cisplatin failed to trigger TRAIL‐induced cell death (Figure 3D and E). Moreover, treatment with NF‐κB activation inhibitor failed to induce TRAIL cytotoxicity in IFN‐γ–treated IMR‐32 cells (Figure 3F). These results indicate that the restoration of caspase‐8 expression in IMR‐32 cells by IFN‐γ treatment failed to induce TRAIL cytotoxicity, although cisplatin increased DR5 expression via downregulation of NF‐κB activity.

### 3.4 Protein kinase B (Akt) or ERK signalling pathway had no role in the susceptibility of IMR‐32 cells to TRAIL

To identify the factors that confer IMR‐32 cells resistance to TRAIL, we examined the expression levels of several molecules related to cell death. As shown in Figure S5, phosphorylated (p‐) Akt (Thr^308^), p‐Akt (Ser^473^) and p‐ERK1/2 were increased by cisplatin treatment, while the expression levels of other molecules such as cellular FLICE‐like inhibitor protein (c‐FLIP) long (c‐FLIP_L_), c‐FLIP short (c‐FLIP_S_), heat‐shock protein (HSP) 70, survivin and Mcl‐1 rarely changed. To inhibit Akt or ERK signalling pathway in these cells, we pretreated them with the chemical inhibitor LY294002 or U0126, respectively, and found that neither LY294002 nor U0126 had any significant effect on cell death induced by cisplatin and/or TRAIL treatment (Figure S6A and B). We obtained similar results in caspase‐8–positive IMR‐32 cells treated with IFN‐γ (Figure S6C and D), indicating that Akt or ERK signalling pathway had no role in the susceptibility of TRAIL‐resistant IMR‐32 cells to TRAIL.

### 3.5 Inhibition of N‐myc expression affected the molecular and cellular characteristics in IMR‐32 cells

Compared to the negative control SK‐N‐MC cells, IMR‐32 and SK‐N‐BE cells showed increased expression of N‐myc (Figure 4A), which was unaltered following cisplatin and/or TRAIL treatment for 24 hours (Figure 4B). In addition, Bcl‐2 family proteins such as Bcl‐2, Bid and Bax were highly expressed in IMR‐32 cells (Figure 4A), and their expression levels were unaffected by cisplatin and/or TRAIL treatment (Figure 4B). Next, we established N‐myc–downregulated IMR‐32 cells using shRNA lentiviral particles targeting *N‐myc* and observed an effective decrease in N‐myc expression in them (Figure 4C). The downregulation of N‐myc expression affected the molecular and cellular characteristics of IMR‐32 cells. Transduction with scrambled shRNA had marginal effect on the morphology of IMR‐32 cells, while N‐myc–downregulated IMR‐32 cells were not colonized and showed some morphological changes such as flat cell bodies (Figure 4D). Moreover, N‐myc–downregulated IMR‐32 cells showed an increase in the expression of p27^Kip1^ (Figure 4E), leading to decreased proliferation in culture (Figure 4F). In addition, N‐myc–downregulated IMR‐32 cells showed a decrease in Bcl‐2 expression (Figure 4E), which could induce a mild collapse of MMP in these cells (Figure 4G). Although N‐myc downregulation failed to restore caspase‐8 expression (Figure 4H), the activation of caspase‐9 and caspase‐3, and cleavage of PARP were slightly increased in IMR‐32 cells (Figure 4I). These results suggest that the inhibition of N‐myc expression can change the characteristics of IMR‐32 cells and increase their susceptibility to cell death.

### 3.6 Inhibition of N‐myc expression sensitized IMR‐32 cells expressing caspase‐8 to TRAIL

**Figure 5:**
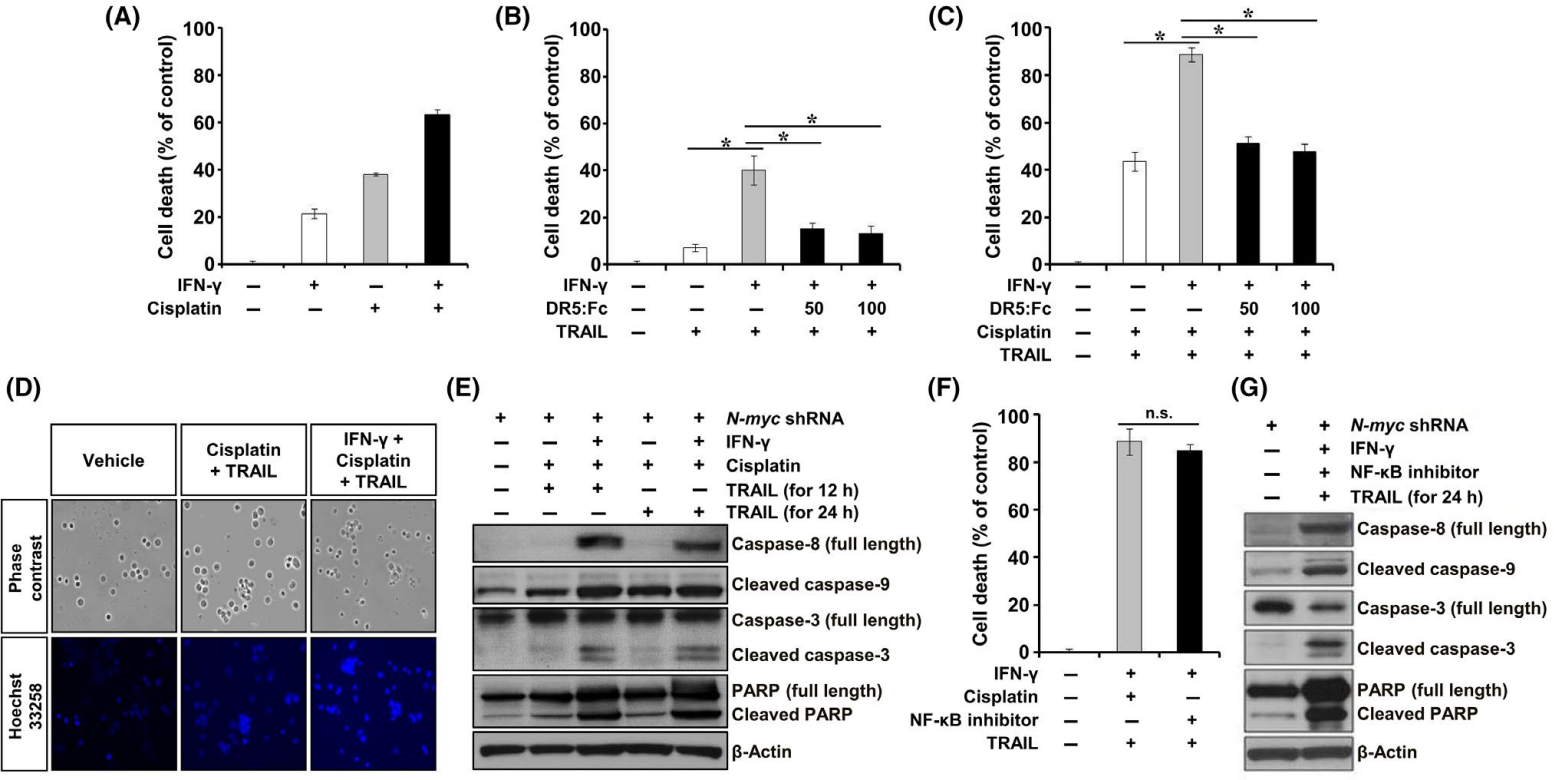
Caspase‐8 expression in N‐myc–downregulated IMR‐32 cells triggered TRAIL cytotoxicity, which was enhanced by cisplatin pretreatment. A, N‐myc–downregulated IMR‐32 cells exposed to 1000 IU/mL IFN‐γ for 24 h, followed by their treatment with 100 ng/mL TRAIL for 24 h. B, N‐myc–downregulated IMR‐32 cells exposed to 1000 IU/mL IFN‐γ for 24 h, followed by their treatment with 100 ng/mL TRAIL for 24 h in the absence or presence of 50 or 100 ng/mL DR5:Fc. C, N‐myc–downregulated IMR‐32 cells exposed to 1000 IU/mL IFN‐γ for 24 h, followed by their treatment with 750 ng/mL cisplatin and 100 ng/mL TRAIL for 24 h in the absence or presence of 50 or 100 ng/mL DR5:Fc. D, Representative images of these cells stained with Hoechst 33258. E, Analysis of caspase‐8 expression and caspase‐9, caspase‐3 and PARP cleavage by immunoblotting. F, N‐myc–downregulated IMR‐32 cell death when exposed to 1000 IU/mL IFN‐γ for 24 h, followed by their treatment with 10 μmol/L NF‐κB inhibitor and 100 ng/mL TRAIL for 24 h, by Alamar Blue assay. G, Analysis of caspase‐8 expression and caspase‐9, caspase‐3 and PARP cleavage by immunoblotting. β‐Actin was used as an internal control. Representative immunoblots are shown. Data are expressed as the percentage (mean ± SD) of vehicle‐treated control cells from three independent experiments. IFN‐γ, interferon‐γ

Next, we examined the effect of N‐myc inhibition on TRAIL susceptibility in IMR‐32 cells. The treatment of myc–downregulated IMR‐32 cells with IFN‐γ to restore caspase‐8 expression resulted in about 20% cell death (Figures 5A and S7), and treatment with IFN‐γ and TRAIL further increased the cell death to about 40% (Figure 5B). In addition, N‐myc–downregulated IMR‐32 cells pretreated with IFN‐γ and subjected to cisplatin and TRAIL co‐treatment showed a dramatic increase in apoptotic cell death (almost 90%), which was accompanied by nuclear condensation and fragmentation, compared to IFN‐γ–untreated cells (Figure 5C and D). Moreover, the activation of caspase‐9 and caspase‐3 and cleavage of PARP significantly increased in these cells (Figure 5E). An additional pretreatment of these cells with DR5:Fc chimera protein effectively abrogated the increased cell death (Figure 5B and C), indicating that the restored TRAIL cytotoxicity induced apoptosis‐mediated cell death. TRAIL cytotoxicity was also observed in IMR‐32 cells that are controlled by N‐myc and caspase‐8 expression, when pretreated with NF‐κB inhibitor (Figure 5F). In addition, the activation of caspase‐9 and caspase‐3 and cleavage of PARP significantly increased in these cells (Figure 5G). These results indicate that the inhibition of N‐myc expression sensitized IMR‐32 cell expressing caspase‐8 to TRAIL, and this effect was dramatically enhanced in the presence of cisplatin, via increased expression of DR5 through the downregulation of NF‐κB activity.

## 4 DISCUSSION

Tumour necrosis factor–related apoptosis‐inducing ligandis widely used for cancer therapy and effectively kills several transformed cells but not normal cells.^12^, ^31^ Although TRAIL may serve as a promising cancer therapeutic agent, many cancer cells exhibit partial or complete resistance to TRAIL.^12^, ^31^ This resistance may be attributed to the deregulated expression of the TRAIL receptors or intracellular components which act downstream of the receptors. Previous studies have shown that the conventional chemotherapeutic agents,^20^, ^32^ irradiation^33^, ^34^ and histone deacetylase inhibitors^35^ enhance the cytotoxicity of TRAIL via upregulation of TRAIL receptors. The upregulation in the expression of DRs by chemotherapeutic agents is dependent on the activity of NF‐κB^36^ or p53,^37^, ^38^ which transcriptionally regulates the expression of DRs by binding to the promoter sites.^39^, ^40^ In particular, the upregulation in DR expression by cisplatin results in an increase in TRAIL‐induced cell death in various cancer cells.^22^, ^23^, ^24^ In this study, cisplatin increased the expression of DR5 in neuroblastoma IMR‐32 cells via downregulation of NF‐κB activity, while increased DR5 levels failed to trigger TRAIL cytotoxicity in TRAIL‐resistant IMR‐32 cells, which showed amplified *MYCN* expression and lacked caspase‐8 expression.

Caspase‐8 is an essential mediator for the initiation of DR‐induced apoptosis^41^, ^42^ and frequently absent in cancers such as neuroblastoma,^41^, ^43^, ^44^ medulloblastoma,^43^, ^45^ small cell lung cancer^46^ and melanoma.^41^ The loss of caspase‐8 expression correlates with low sensitivity to TRAIL cytotoxicity. Cancer cells expressing caspase‐8 are sensitive to TRAIL cytotoxicity, while cells lacking caspase‐8 are TRAIL‐resistant. Moreover, cells lacking caspase‐8 are sensitized to TRAIL cytotoxicity upon restoration of caspase‐8 expression in these cells by 5‐AzaC or IFN‐γ.^41^, ^43^, ^44^, ^45^ The loss of caspase‐8 expression is the result of gene silencing by aberrant methylation and may be restored by treatment with demethylating agents such as AzaC.^43^, ^44^ However, the clinical use of demethylating agents has been limited, owing to their toxic side effects.^47^ IMR‐32 cells used in this study were negative for caspase‐8 expression, and treatment of these cells with IFN‐γ restored their caspase‐8 expression. IFN‐γ restores caspase‐8 expression through its transcriptional activation, which involves signal transducer and activator of transcription 1 (STAT1) or interferon regulatory factor 1 (IRF1) pathway.^45^, ^48^, ^49^ Recent reports showed that IFN‐γ may increase caspase‐8 levels and thereby sensitize tumour cells to apoptotic stimuli.^43^, ^45^, ^49^ In this study, however, IFN‐γ–mediated restoration of caspase‐8 had no effect on TRAIL‐induced cell death, suggestive of the involvement of other factors in TRAIL resistance in IMR‐32 cells.

The neuroblastoma cell line IMR‐32 shows amplification of *MYCN* oncogene and contains more than 25 copies of *MYCN* oncogene per cell.^50^ The amplification of *MYCN* oncogene is one of the most powerful prognostic factors in neuroblastoma,^5^ and its positive effects on tumorigenesis are well known^3^, ^8^; thus, strategies for targeting N‐myc may serve as a promising approach for neuroblastoma therapies.^2^, ^4^ However, recent studies displayed paradoxical results related to the role of N‐myc protein in chemotherapies for the treatment of neuroblastoma.^51^, ^52^, ^53^ The highly expressed N‐myc protein in neuroblastoma increases the sensitivity to chemotherapeutic agents.^51^ Thus, the precise roles of N‐myc protein in chemotherapies are still unclear. In this study, we used retrovirus‐mediated delivery of N‐myc shRNA to downregulate N‐myc protein expression in IMR‐32 cells. TRAIL treatment in these N‐myc–negative cells slightly induced apoptotic cell death, and TRIAL cytotoxicity was increased upon pretreatment of these cells with IFN‐γ. TRAIL cytotoxicity was abrogated by an additional pretreatment with DR5:Fc chimera protein, indicating that the inhibition of N‐myc expression triggered TRAIL cytotoxicity in IMR‐32 cells expressing caspase‐8.

N‐myc protein is generally known to modulate the function of the extrinsic death‐inducing pathway as a molecule downstream of TRAIL receptors as well as the intrinsic apoptosis pathway, which plays a role in maintaining the integrity of the mitochondria via Bcl‐2 family proteins.^54^, ^55^, ^56^ Several reports suggest that Bcl‐2 expression correlates with the expression levels of N‐myc protein, and together, these may promote the survival of lymphoma^57^ and neuroblastoma.^58^ How N‐myc downregulation may lead to decreased Bcl‐2 expression in cancer cells is questionable. Here, we showed that N‐myc protein inhibition in IMR‐32 cells decreased the expression of Bcl‐2, which decreases the stability of MMP, and regulated the activation of caspases to induce apoptosis. Bcl‐2 protein inhibits the release of cytochrome c from mitochondria to the cytosol and regulates the activation of caspases for apoptosis induction.^59^ In addition, many reports showed that the inhibition of Bcl‐2 expression by chemotherapeutic agents increased the sensitivity of cancer cells to TRAIL.^60^, ^61^

Our results demonstrate for the first time that the inhibition of N‐myc expression sensitizes IMR‐32 cells expressing caspase‐8 to TRAIL. The treatment of TRAIL‐resistant IMR‐32 cells with cisplatin resulted in an increased DR5 expression but failed to trigger TRAIL sensitivity. Furthermore, the downregulation of N‐myc expression and restoration of caspase‐8 expression in TRAIL‐resistant IMR‐32 cells may activate TRAIL signalling, thereby inducing apoptotic cell death. Cisplatin pretreatment dramatically enhanced TRAIL cytotoxicity via increased DR5 expression in these cells. In conclusion, our data suggest that the combination therapy of cisplatin and TRAIL is a promising strategy for treating neuroblastoma that is controlled by the expression of N‐myc and caspase‐8, and its use may provide important information for the development of additional potential therapeutic strategies to fight neuroblastoma.

## DISCLOSURE

The authors declare that they have no competing interests.

## AUTHOR CONTRIBUTIONS

This study was designed and supervised by MWL and KHY. Experiments were conducted by DSK, HRK and HJP. Data analysis was conducted by MWL, DSK, JWL, KWS, HHK and KHY. Funding was obtained by MWL and KHY. The manuscript was written by MWL and KHY. All authors have read and approved the final manuscript.
