# Supplementary material for: Inhibition of N‐myc expression sensitizes human neuroblastoma IMR‐32 cells expressing caspase‐8 to TRAIL
Source: Cell Prolif. 2019 Feb 6;52(3):e12577. doi: 10.1111/cpr.12577 (PMC6536445; doi:10.1111/cpr.12577)
Supplement: Supplementary file 1 [file CPR-52-e12577-s001.docx]

**Inhibition of N-myc expression sensitizes human neuroblastoma IMR-32 cells expressing caspase-8 to TRAIL**

Myoung Woo Lee^1^, E-mail: [mwlee77@hanmail.net](mailto:mwlee77@hanmail.net)

Dae Seong Kim^1^, E-mail: [dssmile@hanmail.net](mailto:dssmile@hanmail.net)

Hye Ryung Kim^1^, E-mail: [ryungyo@gmail.com](mailto:ryungyo@gmail.com)

Hyun Jin Park^1^, E-mail: [ever49@hanmail.net](mailto:ever49@hanmail.net)

Ji Won Lee^1^, E-mail: [leejw.lee@samsung.com](mailto:leejw.lee@samsung.com)

Ki Woong Sung^1^, E-mail: [kwsped@skku.edu](mailto:kwsped@skku.edu)

Hong Hoe Koo^1,2^, E-mail: [hhkoo@skku.edu](mailto:hhkoo@skku.edu)

Keon Hee Yoo^1,3,¶^, E-mail: [hema2170@skku.edu](mailto:hema2170@skku.edu)

^1^*Department of Pediatrics, Samsung Medical Center, Sungkyunkwan University School of Medicine, Seoul, Korea*

^2^***Department of Health Sciences and Technology, and*** *^3^****Department of Medical Device Management and Research, SAIHST, Sungkyunkwan University****, Seoul, Korea*

**^¶^Corresponding author: Keon Hee Yoo, MD & PhD**

Department of Pediatrics, Samsung Medical Center, 81 Irwon-ro, Gangnam-gu, Seoul 06351, Korea

E-mail: [hema2170@skku.edu](mailto:hema2170@skku.edu); tel.: +82-2-3410-3532; fax: +82-2-3410-0043.

ORCID ID: 0000-0002-5980-7912

**Supplementary materials**

**Supplementary materials and methods**

**Reagents**

Nuclear factor kappa B (*NF-κB p65*) shRNA lentiviral particles (Cat. No: sc-29410-V﻿) were purchased from Santa Cruz Biotechnology, while antibodies specific for phosphorylated (p-) protein kinase B (Akt [Thr^308^]), p-Akt (Ser^473^), total Akt, p-extracellular signal-regulated kinase (ERK) 1/2, total ERK1/2, heat shock protein (HSP) 70, survivin, and induced myeloid leukemia cell differentiation protein (Mcl-1) were obtained from Cell Signaling Technology. Antibodies specific for cellular FLICE-like inhibitor protein (c-FLIP) long (c-FLIP_L)_, c-FLIP short (c-FLIP_S_), and β-actin were supplied by Santa Cruz Biotechnology. 5-Azacytidine (5-AzaC) was procured from Sigma-Aldrich and U0126 and LY294002, from Calbiochem.

**Cell culture**

Human malignant neuroblastoma cell lines IMR-32 and SK-N-BE, and neuroepithelioma cell line SK-N-MC were purchased from the ATCC. Cells were grown in non-coated T75 culture flasks (Nalgen Nunc), in DMEM, supplemented with 10% FBS and 100 U/mL penicillin/streptomycin (Life Technologies-Gibco), in a humidified 5% CO_2_ atmosphere at 37°C. The medium was changed every 3 days.

**Measurement of human NF-κB (p50/p65) activity**

The DNA-binding activity of NF-κB in cells was quantified by ELISA using NF-κB p50 and p65 transcription factor assay kit (Cayman, Ann Arbor, MI). IMR-32 cells exposed to NF-κB activation inhibitor for 12 h were collected, and their nuclear extract was isolated using the NE-PER nuclear and cytoplasmic extraction reagent kit (Thermo Fisher Scientific). The nuclear extract was quantified with the bicinchoninic acid (BCA) protein assay kit (Pierce Biotechnology, Rockford, IL). A double-stranded oligonucleotide containing a consensus binding site for NF-κB was immobilized in all the wells of a 96-well plate and incubated overnight with the nuclear extracts (7.5 μg/well) at 4°C without shaking. The plate was then extensively washed according to manufacturer’s instructions, and incubated with a primary anti-NF-κB antibody, followed by horseradish peroxidase (HRP) conjugated secondary antibody treatment for detection. The absorbance was expressed as the optical density at 450 nm wavelength and normalized to the background readings.

**Luminex assay**

Caspase-3 activation and PARP cleavage were assessed using 3-Plex apoptosis signaling kit (Merck Millipore, St. Charles, MO) according to the manufacturer’s instructions. Briefly, cells were collected and lysed using the cell signaling universal lysis buffer. The amount of protein was quantified with the BCA protein assay kit and samples containing equal amounts of proteins were incubated overnight in the dark, with a suspension containing beads with capture antibodies specific for active caspase-3, cleaved PARP, and glyceraldehyde-3-phosphate dehydrogenase (GAPDH). The beads were washed and incubated for 1 h with biotinylated reporter antibodies. The beads were washed and treated with streptavidin-PE for 30 min. After washing, the fluorescence intensity of the beads was assessed using a Luminex 200 system (Luminex Corporation, Austin, TX) and analyzed using the MasterPlex QT software (MiraiBio, Alameda, CA). Universal assay buffer was used washing. The fluorescence intensity of GAPDH was used as an internal control.

**Establishment of NF-κB–downregulated IMR-32 cells**

For transduction of IMR-32 cells with NF-κB–targeting lentiviral particles, cells were pretreated with 5 μg/mL polybrene prepared in Dulbecco’s modified Eagle’s medium (DMEM) containing 10% fetal bovine serum (FBS), followed by their incubation with lentivirus particles at a multiplicity of infection (MOI) of 5. After incubation for 24 h in a humidified atmosphere at 37°C with 5% CO_2_, cells were washed twice with phosphate-buffered saline (PBS) and fresh medium was added. Infected cells were selected by incubation for 10 days in the medium containing 5 μg/mL puromycin after 2 weeks of transduction.

**Cell viability: Alamar Blue assay**

Cells (2 × 10^4^ cells/well) were seeded in wells of 96-well plates in 100 μL DMEM without phenol red and supplemented with 1% FBS. Cells were treated for 24-96 h with either 5-AzaC, interferon-γ, U0126, LY294002, cisplatin, or TRAIL separately and in different combinations, as described in the individual experiment. Following treatment, cells were incubated with 11 μL of 1× Alamar Blue® for 3 h and their absorbance recorded at 570 and 600 nm wavelength with an enzyme-linked immunosorbent assay (ELISA) reader.

**Supplementary figures and legends**

**
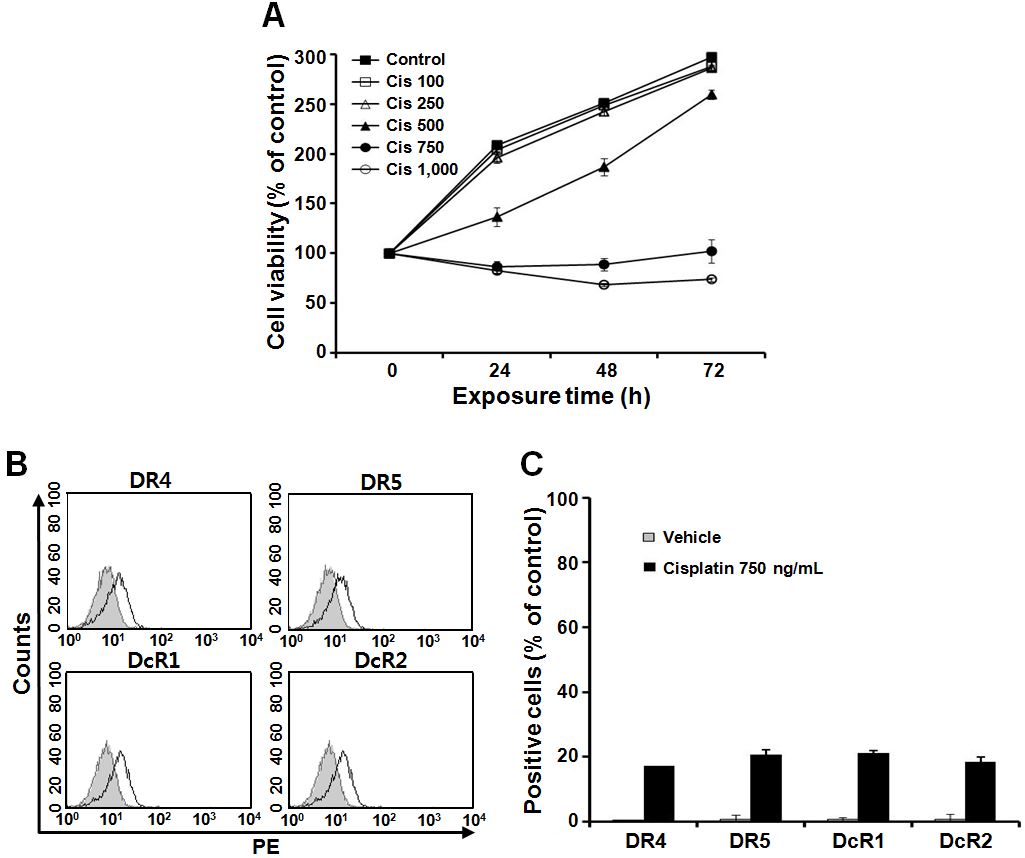
**

**Supplementary Figure 1. Cisplatin treatment induced the expression of TRAIL receptors in SK-N-BE cells.** (**A**) Cell viability in response to treatment with 100, 250, 500, 700, and 1,000 ng/mL cisplatin was analyzed at indicated times in human neuroblastoma SK-N-BE cells. Cell viability was analyzed by Alamar Blue assay. Data are expressed as the percentage of vehicle-treated control cells and represent the mean ± SD from two independent experiments. (**B**, **C**) The expression of TRAIL receptors in SK-N-BE cells was analyzed by flow cytometry using PE-conjugated receptor-specific antibodies for each receptor. Positive cells were plotted against the appropriate IgG isotype controls (shaded histogram). (**B**) Data obtained at 24 h after treatment with vehicle (unshaded gray line) or 750 ng/mL cisplatin (unshaded black line) are presented. (**C**) Changes in the expression of TRAIL receptors in response to cisplatin are shown as a bar graph. Data are expressed as the percentage of IgG isotype controls and represent the mean ± SD from two independent experiments. Cis, cisplatin.


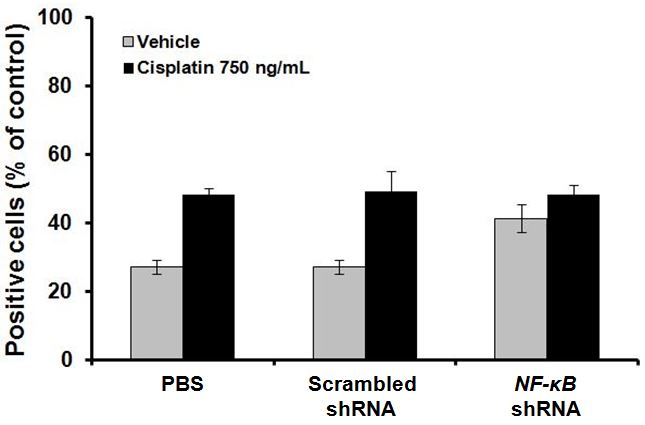


**Supplementary Figure 2. Downregulation of NF-κB p65 expression increased DR5 expression in IMR-32 cells.** The expression of DR5 was analyzed by flow cytometry using PE-conjugated receptor-specific antibody for DR5 in NF-κB–downregulated IMR-32 cells. Positive cells were plotted against the appropriate IgG isotype controls. Data obtained at 24 h after treatment with vehicle or 750 ng/mL cisplatin are presented. Data are expressed as the percentage of IgG isotype controls and represent the mean ± SD from two independent experiments.


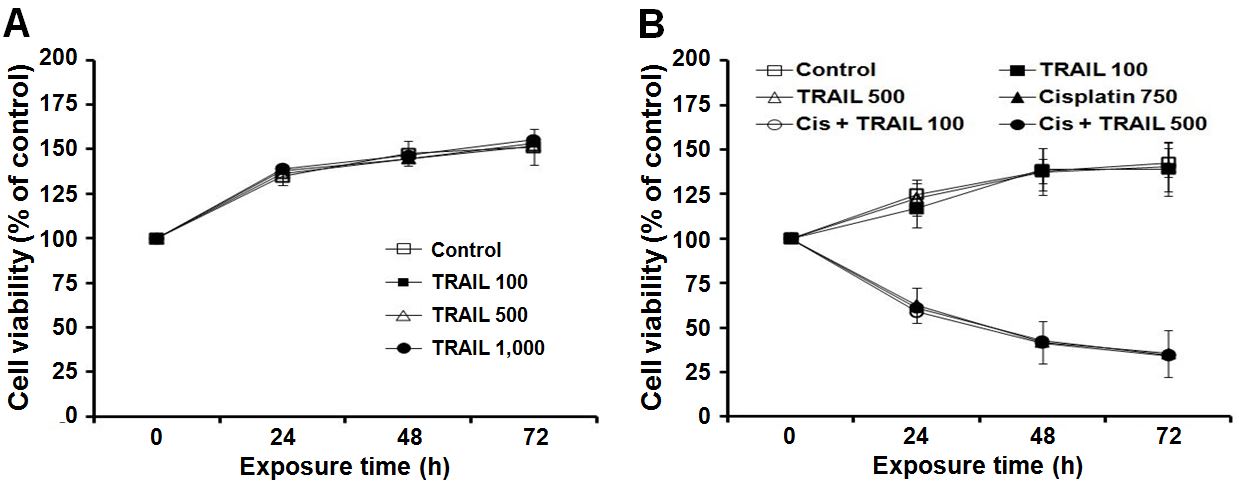


**Supplementary Figure 3. Cisplatin treatment failed to trigger TRAIL cytotoxicity in TRAIL-resistant SK-N-BE cells.** (**A**) Cell viability in response to treatment with 100, 500, and 1,000 ng/mL TRAIL was analyzed at indicated times. (**B**) Cell viability in response to pretreatment with 750 ng/mL cisplatin for 12 h and consecutive treatment with 100 or 500 ng/mL TRAIL was analyzed at indicated times. Cell viability was analyzed by Alamar Blue assay. Data are expressed as the percentage of vehicle-treated control cells and represent the mean ± SD from two independent experiments. Cis, cisplatin.


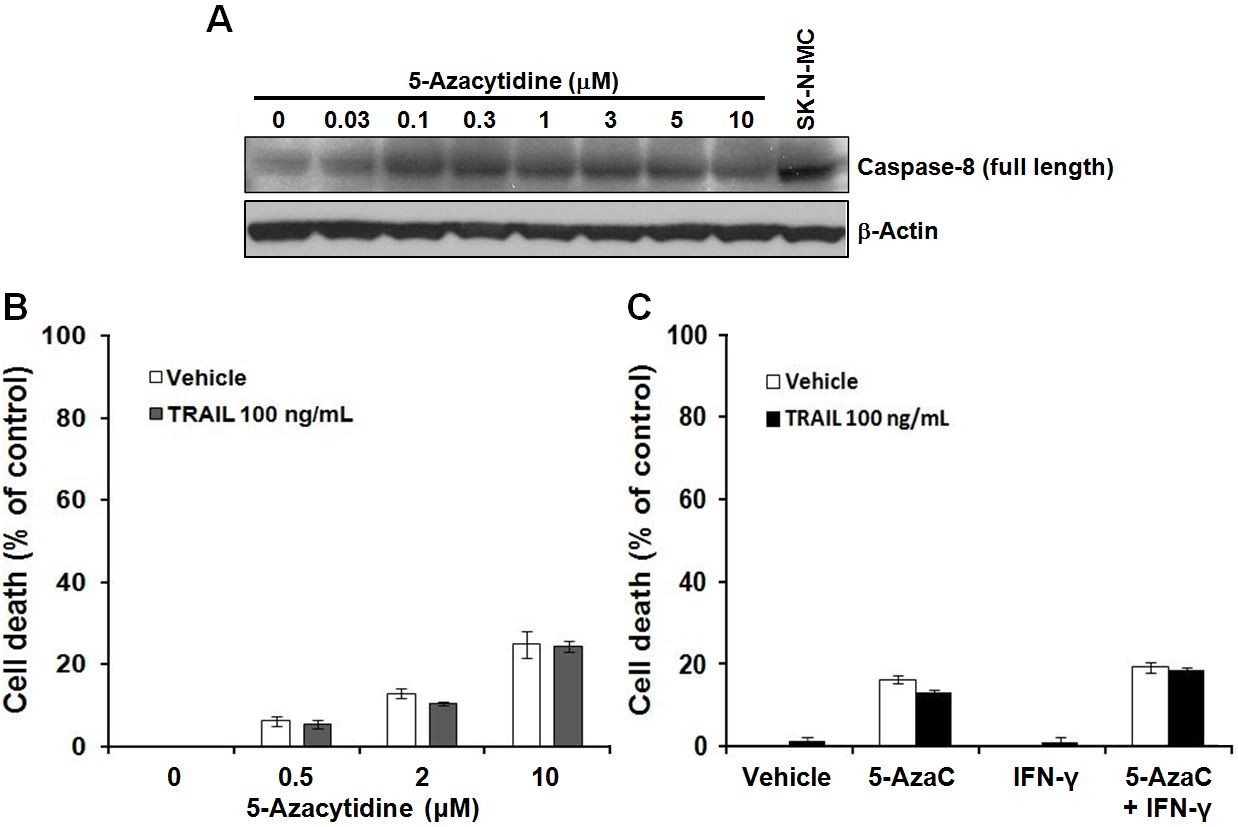


**Supplementary Figure 4. 5-Azacytidine treatment restored caspase-8 expression but failed to trigger TRAIL cytotoxicity in IMR-32 cells.** (**A**) Caspase-8 expression induced by treatment with the indicated concentrations of 5-AzaC for 24 h in IMR-32 cells. SK-N-MC cells were used as a positive control. (**B**) IMR-32 cells were exposed to the indicated concentrations of 5-AzaC for 24 h, followed by their treatment with 100 ng/mL TRAIL for 48 h. (**C**) IMR-32 cells were exposed to 2 μM 5-AzaC and/or 1,000 IU/mL IFN-γ for 24 h, followed by their treatment with 100 ng/mL TRAIL for 48 h. Cell death was analyzed by Alamar Blue assay. Data are expressed as the percentage of vehicle-treated control cells and represent the mean ± SD from two independent experiments. 5-AzaC, 5-azacytidine; IFN-γ, interferon-γ.


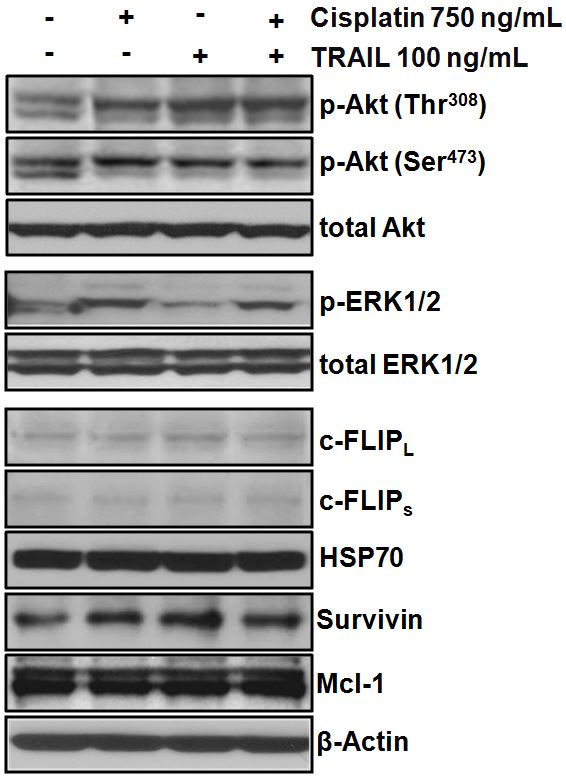


**Supplementary Figure 5. Immunoblotting analysis to evaluate the changes in the protein levels in IMR-32 cells.** IMR-32 cells pretreated with 750 ng/mL cisplatin for 12 h, followed by their treatment with 100 ng/mL TRAIL for 24 h. Data show the protein levels of p-Akt (Thr308), p-Akt (Ser473), total Akt, p-ERK1/2, total ERK, c-FLIP_L_, c-FLIP_S_, HSP70, survivin, and Mcl-1. β-Actin was used as an internal control. Representative immunoblots are shown.


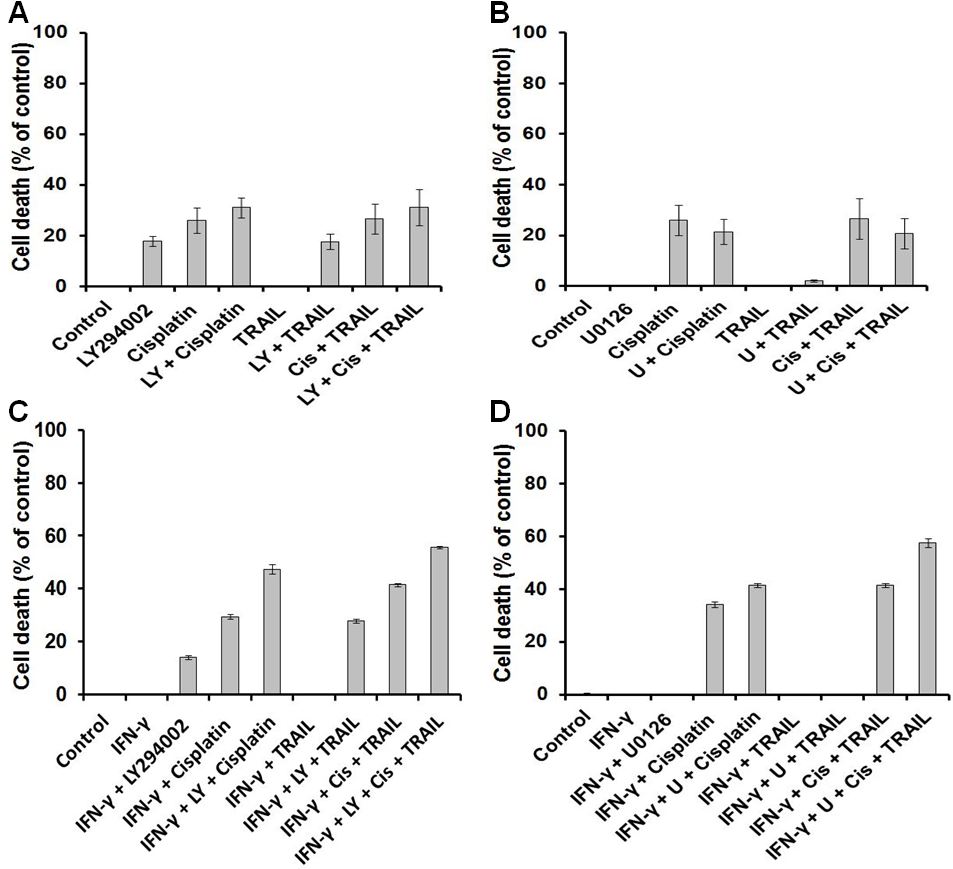


**Supplementary Figure 6. Inhibition of Akt or ERK signaling failed to trigger TRAIL cytotoxicity in IMR-32 cells.** (**A**) Cell death in response to pretreatment with 10 μM LY294002 for 24 h, followed by treatment with 750 ng/mL cisplatin and/or 100 ng/mL TRAIL for 48 h was analyzed by Alamar Blue assay. (**B**) Cell death in response to pretreatment with 1 μM U0126 for 24 h, followed by treatment with 750 ng/mL cisplatin and/or 100 ng/mL TRAIL for 48 h was analyzed by Alamar Blue assay. (**C**) Cell death in response to pretreatment with 1,000 IU/mL IFN-γ and/or 10 μM LY294002 for 24 h, followed by treatment with 750 ng/mL cisplatin and/or 100 ng/mL TRAIL for 48 h was analyzed by Alamar Blue assay. (**D**) Cell death in response to pretreatment with 1,000 IU/mL IFN-γ and/or 1 μM U0126 for 24 h, followed by treatment with 750 ng/mL cisplatin and/or 100 ng/mL TRAIL for 48 h was analyzed by Alamar Blue assay. Data are expressed as the percentage of vehicle-treated control cells and represent the mean ± SD from two independent experiments. LY, LY294002; Cis, cisplatin; U, U0126; IFN-γ, interferon-γ.


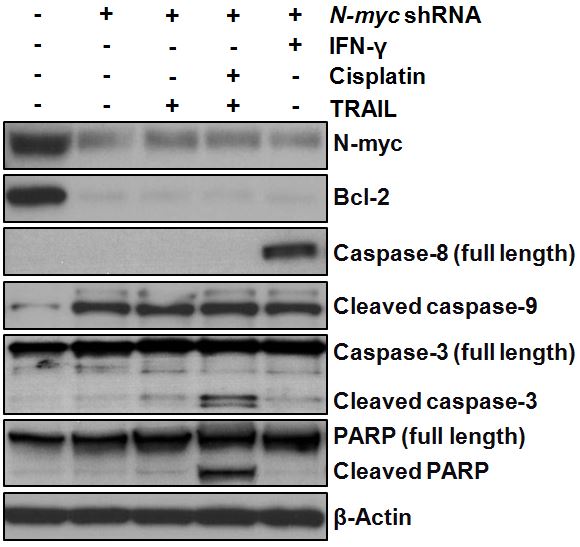


**Supplementary Figure 7. Immunoblotting analysis to evaluate the changes in the protein levels in N-myc–downregulated IMR-32 cells.** N-myc–downregulated IMR-32 cells were exposed to either 750 ng/mL cisplatin and/or 100 ng/mL TRAIL or 1,000 IU/mL IFN-γ for 24 h. N-myc, Bcl-2, and caspase-8 expression and caspase-9, caspase-3, and PARP cleavage were detected by immunoblotting. β-Actin was used as an internal control. Representative immunoblots are shown. IFN-γ, interferon-γ.
